# Exploring the Potential to Repurpose Flexible Moulded Polyurethane Foams as Acoustic Insulators

**DOI:** 10.3390/polym14010163

**Published:** 2021-12-31

**Authors:** Enikő Mester, Dániel Pecsmány, Károly Jálics, Ádám Filep, Miklós Varga, Kitti Gráczer, Béla Viskolcz, Béla Fiser

**Affiliations:** 1 Institute of Chemistry, University of Miskolc, 3515 Miskolc, Hungary; kemeniko@uni-miskolc.hu (E.M.); kemdanip@uni-miskolc.hu (D.P.); kemmiki@uni-miskolc.hu (M.V.); kitti.graczer@uni-miskolc.hu (K.G.); bela.viskolcz@uni-miskolc.hu (B.V.); 2 Higher Education and Industrial Cooperation Centre, University of Miskolc, 3515 Miskolc, Hungary; femfilep@uni-miskolc.hu; 3 Department of Machine Elements, University of Miskolc, 3515 Miskolc, Hungary; machijk@uni-miskolc.hu; 4 Institute of Physical Metallurgy and Metal Forming, University of Miskolc, 3515 Miskolc, Hungary; 5 Ferenc Rákóczi II, Transcarpathian Hungarian College of Higher Education, 90200 Beregszász, Transcarpathia, Ukraine

**Keywords:** polyurethane repurposing, flexible foam, compressive strength, sound insulation, waste utilization

## Abstract

Polyurethane flexible foams are widely used for a variety of applications to improve comfort and durability. Their long-term frequent use inevitably leads to the generation of waste that needs to be treated. The recycling and reuse of polyurethane waste are essential to achieve an environmentally friendly economy. The present study investigates the potential to reuse and repurpose flexible polyurethane foam from automotive seat cushion waste materials. Flexible foams were prepared with different hardnesses using isocyanate–polyol ratios between 0.8 and 1.2 NCO-index. Dry heat aging tests were performed to mimic the long-term usage of the materials. The decrease in compressive strength was compared with the change in acoustic damping properties before and after the aging tests using an acoustic tube, and the change in foam cell structures was also analyzed by micro-CT. On the basis of the results obtained, although the foam systems are no longer suitable to be used as seat cushions due to aging, they can still be used as sound insulation materials within a given frequency range, as their sound absorption capacity is suitable for such purpose.

## 1. Introduction

Flexible polyurethane (PU) foams are complex porous materials widely used in several comfort applications such as automotive seats and other types of cushioning [1]. There are two main methods for their industrial production: a continuous slabstock production process and a discontinuous molded process [2]. The material quality of flexible PU foams depends on their composition. The two main raw materials are isocyanate and polyol, which together form urethane bonds during polymerization, in addition to which suitable additives such as catalyst, blowing agent, and surfactant must be used to create a stable foam of adequate quality [3]. The polymeric phase can be considered as a block copolymer comprising a soft continuous matrix of polyether segments with dispersed hard segments consisting of urethane and urea groups [4]. The number of hard segments is based on the isocyanate ratio [5]. Hydrogen bonds in this segment are in part crystalline and act as physical crosslinks in the polymer [6].

Polyurethane foam is characterized not only by its solid-phase chemical structure but also by its morphology. It has already been proven that the different mechanical properties are majorly influenced by the relative density, cell sizes, and anisotropy of the foam [7]. Following technological development, comfort has become an important factor along with the mechanical, vibrational, and acoustic characteristics of the foam [8]. As a result, the improvement of the sound absorption capacity of polyurethane foam-based products has become inevitable to ensure a comfortable ride by minimizing indoor noise [9]. This is especially important due to the rise in electric cars, as the slightest noise can be heard due to the lack of engine noise [10]. It is known that polyurethane is a highly damping material for acoustic vibrations in the high frequency range. However, due to its elastic nature in the lower-frequency range (around 1 Hz), the vibration damping is limited [11]. There are two main mechanisms which play a role in acoustic attenuation. These foams are considered to be an open-cell structure, because the pores formed by the gas bubbles interconnect without barriers between them; the solid matrix structure between the bubbles is a system of ligaments [12]. It is known that sound energy can be absorbed by the friction between gas molecules of air oscillating inside the cells of the foam [13]. As a result of this phenomenon, kinetic energy transforms into heat, where the cell morphology is primarily responsible for the amount of sound energy absorbed. If the material pores are too large, the sound wave will pass through it easily, which causes minimal acoustic dampening. If the porosity is overly fine, i.e., the cells are too small, the majority of the energy will reflect back into the environment from the surrounding region of the surface, never entering deeply enough to cause the significant absorption of sound waves [12]. Moreover, the material of the cell wall can absorb sound in what is known as intrinsic damping, whereby the sound waves propagate inside the material [7]. Imai and Asano concluded that the main factor influencing sound absorption of flexible polyurethane foams was the flow resistance properties determined by airflow measurements [14]. Furthermore, typically, the sound absorption coefficient is mainly associated with the morphological properties of the polyurethane foam [15]. The dynamic mechanical properties and acoustic damping, as well as the optimization of performance-to-weight ratio, of flexible PU foams for automotive seating applications have been studied before [13]. Altering the chemical structure of the solid phase of the polyurethane foams or adding various kinds of additives to the mixture are some of the known ways to modify the performance of the products [16]. For this reason, by modifying the isocyanate–polyol ratio, the ratio of hard to soft segments also changes, which changes the mechanical properties. Overall, scientific studies have so far concentrated on obtaining the highest sound absorption coefficient at specific samples. Mosanenzadeh et al. [17] examined poly(lactic acid) open-cell foams, in which case average cell sizes between 250 and 500 μm revealed the best sound absorption performance. Kuranska et al. [18] measured the sound absorption coefficient of PU foams with different average cell sizes (diameters) from 581 ± 143 µm to 2936 ±153 µm and concluded that, in terms of acoustic properties, the materials with large cell sizes exhibited better sound absorption, especially in the frequency range above 400 Hz. Nevertheless, obtaining high sound absorption performance in a wide range of samples is critical to guarantee industrial interest with established reproducibility [5].

However, the sound absorption feature of flexible foams has not been applied in repurposing polyurethane-based materials. Through the continual improvement in environmental awareness, especially in today’s society, people are increasingly realizing that resources are not endless [19]. Polyurethane foam waste must be disposed of and recycled efficiently, which is not just a requirement to avoid pollution and to protect the environment, but also to reduce production costs and improve material use. Due to its low density and large volume, polyurethane foam waste is difficult to handle, while its combustion produces toxic gases [20]. There are two ways to recycle polyurethane foam waste: physical recycling and chemical recycling [21]. Chemical recycling follows the principle of degradation. Polyurethane wastes are gradually depolymerized to the original reactants or other oligomeric or small-molecule organic compounds [3,22,23]. Physical recycling is the direct reuse of polyurethane waste without chemical treatment. In the present study, a potential physical recycling method of flexible PU foams used in car seat cushions is proposed on the basis of their sound absorption properties.

## 2. Materials and Methods

### 2.1. Materials

Polyurethane flexible foams were produced using a high-pressure polyurethane reaction casting machine (Topline KH by Hennecke GmbH, Sankt Augustin, Germany). The precursors for the production were Ongronat TR4040 (methylene–diphenyl-diisocyanate isomer mixture, Wanhua-BorsodChem, Kazincbarcika, Hungary) and Ongropur FFP-303 (polyether type polyol premix, Wanhua-BorsodChem, Kazincbarcika, Hungary). The polyol system already contains catalysts and other additives such as blowing agents and surfactants which were mixed into a base polyol (Alcupol F2831, Repsol, Madrid, Spain) since the equipment does not contain a separate additive circuit. Samples with different raw material ratios and, thus, different hardness were prepared. The raw material ratios (isocyanate index, NCO) were 0.9, 1.0, 1.1, and 1.2 isocyanate to 1.0 polyol. The hardness of the samples increased with increasing isocyanate index due to the greater proportion of hard segments.

### 2.2. Flexible Polyurethane Foam Production

**Figure 1:**
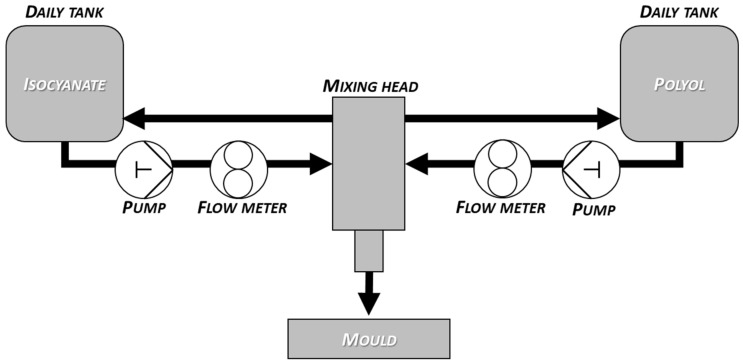
The simplified process flow diagram of flexible molded polyurethane foam production.

The samples were produced with a high-pressure reaction casting machine (Topline KH by Hennecke GmbH, Sankt Augustin, Germany). The equipment is mainly suitable for the production of molded polyurethane soft foam elements, but it is also possible to produce other types of polyurethane systems within the given raw material ratios and machine parameters. Production was carried out in a 400 × 400 × 100 mold. The mixing of the components takes place in the mixing head with a counter-current injection process. The dosing is carried out in thermostated aluminum molds in the mold-carriers (FRIMO Group GmbH, Lotte, Germany) using an ABB robot arm with a high-pressure mixing head. After the injection, the mold carrier closes for the time of the foaming reaction. Proper mold filling is carried out by the eccentric movement of the mold carriers. Upon foaming, the air cushions are released, allowing the carbon dioxide generated during the reaction to escape, which can be done during the reaction if necessary. During production, any other form-bearing movement can be set on the human–machine interface (HMI, Frimo Group GmbH, Lotte, Germany). At the end of the process, the mold-carrier opens, and the finished sample can be removed. Molds need to be treated with a release agent at intervals to make it easier to remove the sample before production begins [24]. The simplified process flow diagram for flexible polyurethane foam production is shown in Figure 1.

### 2.3. Mechanical Measurements

Test Method C of ASTM D3574 [25] (complex test standard for flexible polyurethanes) was followed with minor changes during the mechanical and aging measurements. Cylinders with a diameter of 30 mm were cut out along the total sample height (~95 mm), and then divided into two equal parts along their height. The samples were pressure-tested on their entire surface up to 50% of their height, held there for 1 min, and then the change in force was measured. The value obtained at the end is called compression force deflection (CFD). The measurement was carried out before and after aging to simulate the effect of the long-term usage of the material. The extent of the change is determined by the change in the pressure depth–force curve (force decrease). If the rate of change is too large, the material can no longer be used for compressive stress.

### 2.4. Aging Tests

To determine the effect of long-term use in both mechanical (compressive) stress and sound insulation properties, a standard aging test was carried out. The ASTM D3574 standard contains three different test methods for aging, among which Aging Test K was applied, which is a dry heat aging. During this, the samples were aged in an oven (POL-EKO Aquaterra SLW240 Ventilated Air Mixer Laboratory Oven, Aqua-Terra Lab Ltd., Veszprém, Hungary) for 23 h at 140 °C. After at least 2 h in specified conditions (23 ± 2 °C, 50% ± 5% relative humidity), the mechanical and acoustic values were measured.

### 2.5. Micro-CT Measurements

To examine the microstructural properties, micro-CT (X-ray micro-computed tomography) measurements were also carried out on the samples. The experiments were performed on a YXlon FF35 dual-tube micro-CT device (3D Laboratory, University of Miskolc, Miskolc, Hungary), which evaluated the cell structure using the VG Studio Max module. The device uses a transmission microfocus X-ray source (up to 190 kV and 15 W) with a nominal spot size of 2 µm. The sensor size is 2176 × 1792 pixels. The samples were tested at 60 kV and 7.8 W. The resulting voxel size (i.e., the smallest detectable size) was 7.5 µm. For the tests, a log with a side length of about 8 mm was cut from the middle of the sample volume at the full height of an optimal weight foam (~95 mm); however, since the total sample height could not be measured at the same time due to equipment limitations, the logs were cut into two nearly identical parts along their height.

### 2.6. Acoustic Measurements

To investigate the acoustic damping properties of the prepared flexible polyurethane foams in the higher-frequency range, the normal incident sound absorption coefficients (α) were determined using the impedance tube technique. For the measurements, the AED 1000 AcoustiTube^®^ impedance tube was used, which is a laboratory measurement system for the determination of sound absorption, reflection coefficient, and impedance of test samples according to the transmission function method described in EN ISO 10534–2 [26] and ASTM E1050 [27]. The impedance tube can be supplemented if required with a module that allows the determination of additional acoustic parameters of the sound-absorbing material and the measurement of the sound transmission coefficient (τ) of open-pore materials according to ASTM E2611 (standard test method for normal incidence determination of porous material acoustical properties based on the transfer matrix method) [28]. The frequency range of the measurement is limited by the geometry of the impedance tube and the distances between the microphones. The necessary conditions according to the EN ISO 10534–2 are the following:

Lower frequency limit f_1_:

(1) $$f_{l}=0.05\frac{c}{s};$$

Upper frequency limit f_u_:

(2) $$f_{u,1}\;\cdot \;d<0.58\;\cdot \;c,$$

(3) $$f_{u,2}\;\cdot \;s<0.45\;\cdot \;c,$$

(4) $$f_{u}=\min\left(f_{u,1};f_{u,2}\right).$$

In both cases, d is the diameter of the impedance tube (in m), c is the speed of sound (in m/s), and s is the distance between the microphones (in m). In this study, tubes with an internal diameter of 30 mm were used for the measurements, in which case the useful frequency range was 150–6600 Hz. It is also possible to use the aforementioned extension and, thus, to measure the sound transmission factor. According to the standard for performing the measurements, at least two microphones are needed. In this case, three microphones were used; thus, the measurements for the lower- and upper-frequency range could be performed in a single measurement. The sound pressures were measured with the microphones, and the transfer functions between them were determined as follows:

(5) $$r=\frac{H_{12}-H_{l}}{H_{R}-H_{12}}e^{2{\mathrm{jk}}_{0}x_{1}},$$

where H_12_ is the transfer function between microphone 1 and microphone 2, H_R_ is the transfer function of the reflected wave, H_I_ is the transfer function of the incident wave, k_0_ is the wave number, and x_1_ is the distance between the sample and the farthest microphone (in use). On the basis of this equation, the normal incidence sound absorption coefficient (α) can be calculated as follows:

(6) $$\alpha =1-{\left|r\right|}^{2}.$$

Cylinders with a diameter of 30 mm were cut out along the total sample height (~95 mm), and then divided into three equal parts along their height. The acoustic measurement was carried out before and after aging to determine the effect of long-term sound insulation use of the samples.

## 3. Results and Discussion

### 3.1. Mechanical Tests

A compression force deflection test was carried out according to ASTM D3574 Test C on three samples for each NCO-index (0.8, 0.9, 1.0, 1.1, and 1.2). The force was measured at 50% of the original sample height compression after 1 min. The measurements were carried out twice before and after dry heat aging (ASTM D3574 Test K). The force and height reduction were calculated after aging (Table 1).

**Figure 2:**
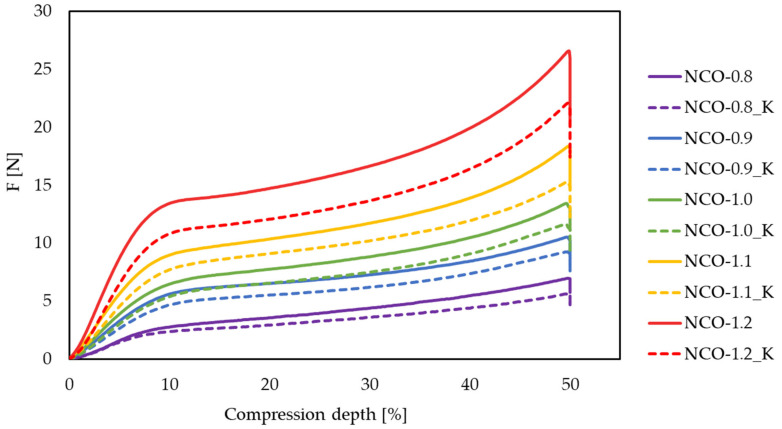
Average compression force (F) deflection curves measured before and after aging. The NCO-index of the samples varied from 0.8 to 1.2. The average curves of the samples after dry heat aging are marked with K (e.g., NCO-0.8_K).

The average compression depth–force curves were recorded for each NCO-index (Figure 2). As the hardness of the foams increased with increasing NCO-index, given that the hard segments were determined by the amount of isocyanate, the compressive force (F) also gradually increased from an average of 5.69 N for an NCO index of 0.8 to 20.67 N in case of the samples with 1.2 NCO-index.

After the aging tests, it appears that the rate of reduction in the compressive strength varied with different hardnesses from an average of 12.65% to 17.67%. The most significant change occurred in the case of the softest (0.8 NCO-index) and the hardest foams (1.2 NCO-index), while the lowest change in load capacity after aging occurred in the case of the 0.9 NCO-index sample. This means that, if the lower load capacity is required, a sample with 0.9 NCO-index is recommended for durability, whereas, if a higher load capacity is required, a sample with 1.2 NCO-index is recommended. Furthermore, if the sample may be exposed to higher temperatures, a significant reduction in load capacity may occur. A force reduction of more than 10% is considered significant in all cases, as these materials are typically designed for a given load capacity. The change in sample heights was very small, <1% after aging in each case, but this indicated a slight expansion of the foam cells.

### 3.2. Results of the Micro-CT Measurements

**Figure 3:**
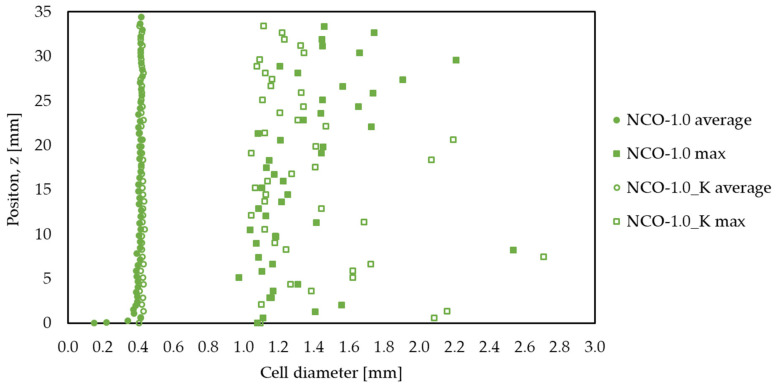
Average and maximum cell diameters along the sample height of the NCO-1.0 sample examined with a micro-CT before (NCO-1.0, filled circles and squares) and after (NCO-1.0_K, empty circles and squares) dry heat aging.

**Figure 4:**
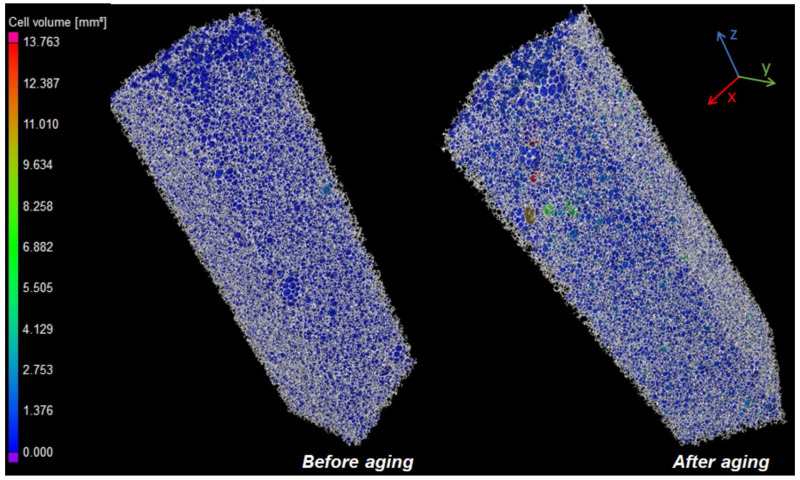
Cell structure of the NCO-1.0 sample examined with a micro-CT before and after dry heat aging.

Using micro-CT measurement, the change in foam cell structure was also examined for all raw material ratios, and the results were found to be very similar for all hardnesses. Therefore, only the sample with NCO-1.0 is presented here (Figure 3 and Figure 4), while the remaining results can be seen in the Supplementary Materials (Figures S1–S4).

The average and maximum cell diameters along the sample height before and after dry heat aging tests were measured. Only a slight increase was observed for the average cell diameters (from 0.40 mm to 0.42 mm), especially near the foam skin layer; however, for the maximum cell diameters, significantly larger cell sizes could be seen, indicating damage to the cell walls. This phenomenon also caused a slight increase in average diameters. The sphericity of the cells was also calculated by the ratio of the sphere surface to the cell surface, which showed no significant difference; in both cases, an average value of 0.57 was obtained.

Extraordinarily large cells can also be observed in the case of the sample after aging with a volume of up to 13.76 mm^3^, which corresponds to a diameter of 2.97 mm if the cell is considered a perfect sphere. This supports that cell-wall damage may have occurred during aging.

### 3.3. Acoustic Measurements

**Figure 5:**
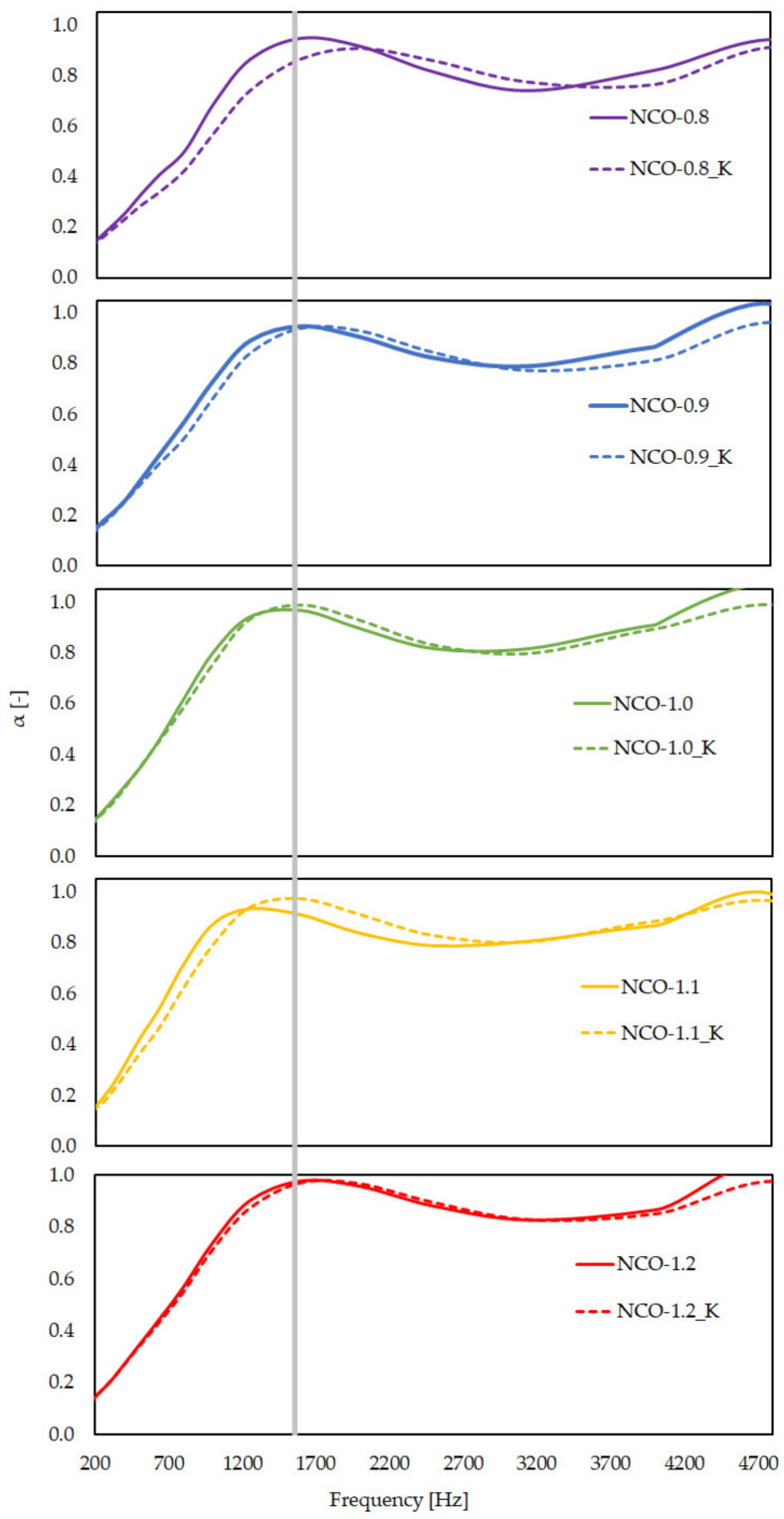
Average curves of the sound absorption coefficient (α). The samples were tested before and after aging, and the NCO index varied from 0.8 to 1.2. The average curves of the samples after dry heat aging are marked with K. The gray line marks the local maximum (~1300 Hz) from which the useful frequency range begins.

The acoustic properties of each sample were measured twice, before and after aging (Figure 5). The samples behaved as expected from a porous sound-absorbing material when measured (on both sides), both before and after aging. The sound absorption coefficient (α) ran evenly and then reached a maximum, before fluctuating, but it remained around 1. Samples before and after aging behaved similarly to each other. There was a local maximum around 1300 Hz, after which the value of the absorption coefficient dropped to ~0.7 and increased back to 1.0 at higher frequencies. 

This is caused by the so-called “skin effect”. The elasticity of the skin (outer denser layer of the foam) of the samples as a mass and the underlying foam form a mass–spring system. At the natural frequency, the abovementioned local maximum occurs; thus, a resonance-type sound absorption dominates. At higher frequencies, the relatively dense skin has high flow resistance; thus, sound waves can penetrate it to a lesser extent, and the underlying foam cannot exert the sound-absorbing effect to the same extent as without the “skin”. During the measurement, the sample was placed in the tube and struck by a steel piston. The effect described above, therefore, only occurred when the “skin” was away from the piston. That is, if the sample is inverted, the “skin” would slide on the plunger, as if it were not present; thus, it would have no effect. Thus, if the sample is inverted, it behaves like the sample after aging, where there is no skin surface at all. After the aging tests, the maximum of the sound absorption coefficient shifted to a very small extent for all NCO indices, appearing at a slightly higher frequency. In the case of the sample with an NCO index of 0.8, the skin effect after aging was not so pronounced, which may have been due to the cell wall damage caused by the higher heat exposure near the skin, which resulted in easier penetration of sound waves. In the case of samples with higher NCO-indices, this effect was very small; thus, it did not affect the sound absorption capacity. Accordingly, in a given frequency band, the samples still have adequate acoustic properties.

## 4. Conclusions

The compressive strength, sound absorption capacity, and foam cell microstructure of flexible polyurethane foam samples with different NCO indices from 0.8 to 1.2 were tested before and after dry heat aging using standard methods. Although some level of cell damage occurred, the average cell size did not change significantly. However, a change of more than 10% in compressive strength was observed in all cases. This was probably due to the fact that the solid “matrix” part of the foam structure was also damaged during the aging process, which greatly affected the mechanical properties. However, the sound absorption capacity of the materials, whose values indicated good insulating properties, did not change significantly. In light of all this, it can be concluded that, when the foams can no longer be used for seating applications, they can still be used as a sound insulating material. Thus, a new possible way of repurposing polyurethane flexible foam seat cushion waste was proposed. The reusing process for these foams is simple; if the sound to be absorbed is in the given frequency range, it is adequate to only cut the samples to the desired shape, and no other additional preparation step is required. Therefore, an easy and cheap way to repurpose and reuse polyurethane waste is achieved.

## Figures and Tables

**Table 1:** Compression force deflection test results according to ASTM D3574 Test C. The first sample with 0.8 NCO-index is indicated as NCO-0.8_1 and NCO-0.8_1_K before and after dry heat aging, respectively. F_50%,0_ is the force measured at 50% sample height compression depth before aging; F_50%,1_ is the force measured at 50% sample height compression depth after aging; h_0_ is the original sample height; h_1_ is the sample height after aging; ΔF_50%_ is the rate of force reduction; Δh is the reduction in the sample height.

NCO-Index	Original Samples	F_50%,0_ (N)	h_0_ (mm)	Samples after Aging	F_50%,1_ (N)	h_1_ (mm)	ΔF_50%_ (%)	Δh (%)	ΔF_50__%, Average_ (%)
0.8	NCO-0.8_1	5.76	33.10	NCO-0.8_1_K	4.49	33.00	22.14	0.29	17.67
	NCO-0.8_2	5.74	34.00	NCO-0.8_2_K	4.91	33.80	14.49	−0.29	
	NCO-0.8_3	5.57	33.99	NCO-0.8_3_K	4.66	33.69	16.39	0.60	
0.9	NCO-0.9_1	8.93	34.48	NCO-0.9_1_K	7.48	34.48	16.21	0.00	
	NCO-0.9_2	9.15	34.04	NCO-0.9_2_K	8.11	34.12	11.36	−0.24	12.65
	NCO-0.9_3	8.11	34.03	NCO-0.9_3_K	7.26	34.03	10.39	−0.01	
1.0	NCO-1.0_1	11.09	34.90	NCO-1.0_1_K	9.46	35.07	14.77	−0.51	14.66
	NCO-1.0_2	11.00	34.27	NCO-1.0_2_K	9.19	34.43	16.49	−0.45	
	NCO-1.0_3	11.21	34.24	NCO-1.0_3_K	9.78	34.39	12.73	−0.44	
1.1	NCO-1.1_1	14.90	33.79	NCO-1.1_1_K	12.48	33.83	16.24	−0.12	16.72
	NCO-1.1_2	15.08	33.99	NCO-1.1_2_K	12.52	34.23	16.99	−0.70	
	NCO-1.1_3	14.08	33.92	NCO-1.1_3_K	11.70	33.94	16.93	−0.05	
1.2	NCO-1.2_1	20.20	33.90	NCO-1.2_1_K	16.90	33.94	16.33	−0.13	17.42
	NCO-1.2_2	21.36	33.84	NCO-1.2_2_K	17.60	33.78	17.63	0.16	
	NCO-1.2_3	20.44	34.05	NCO-1.2_3_K	16.70	34.12	18.30	−0.19	

## Data Availability

Additional figures are available in the supporting information.

## Supplementary Materials

The following are available online at https://www.mdpi.com/article/10.3390/polym14010163/s1: Figures S1–S4: Average and maximum cell diameters along the sample height of the NCO-0.8, NCO-0.9, NCO-1.1, and NCO-1.2 samples, respectively, before and after dry heat aging.
