# Supplementary material for: Exploring the Potential to Repurpose Flexible Moulded Polyurethane Foams as Acoustic Insulators
Source: Polymers (Basel). 2021 Dec 31;14(1):163. doi: 10.3390/polym14010163 (PMC8747745; doi:10.3390/polym14010163)
Supplement: Supplementary file 1 [file polymers-14-00163-s001.zip › polymers-1509147-supplementary.pdf]

polymers

Article

Exploring the Potential to Repurpose Flexible Moulded Polyurethane Foams as Acoustic Insulators

Enikő Mester[1,2], Dániel Pecsmány[1,2], Károly Jálics[3], Ádám Filep[2,4], Miklós Varga[1,2], Kitti Gráczer[1,2], Béla Viskolcz[1,2], Béla Fiser[1,2,5,*]

[1] Institute of Chemistry, University of Miskolc, 3515 Miskolc-Egyetemváros, Hungary
[2] Higher Education and Industrial Cooperation Centre, University of Miskolc, 3515 Miskolc-Egyetemváros, Hungary
[3] Department of Machine Elements, University of Miskolc, 3515 Miskolc-Egyetemváros, Hungary
[4] Institute of Physical Metallurgy and Metal Forming, University of Miskolc, 3515 Miskolc-Egyetemváros, Hungary,
[5] Ferenc Rákóczi II. Transcarpathian Hungarian College of Higher Education, 90200 Beregszász, Transcarpathia, Ukraine,
[*] Correspondence: kemfiser@uni-miskolc.hu

Supplementary information

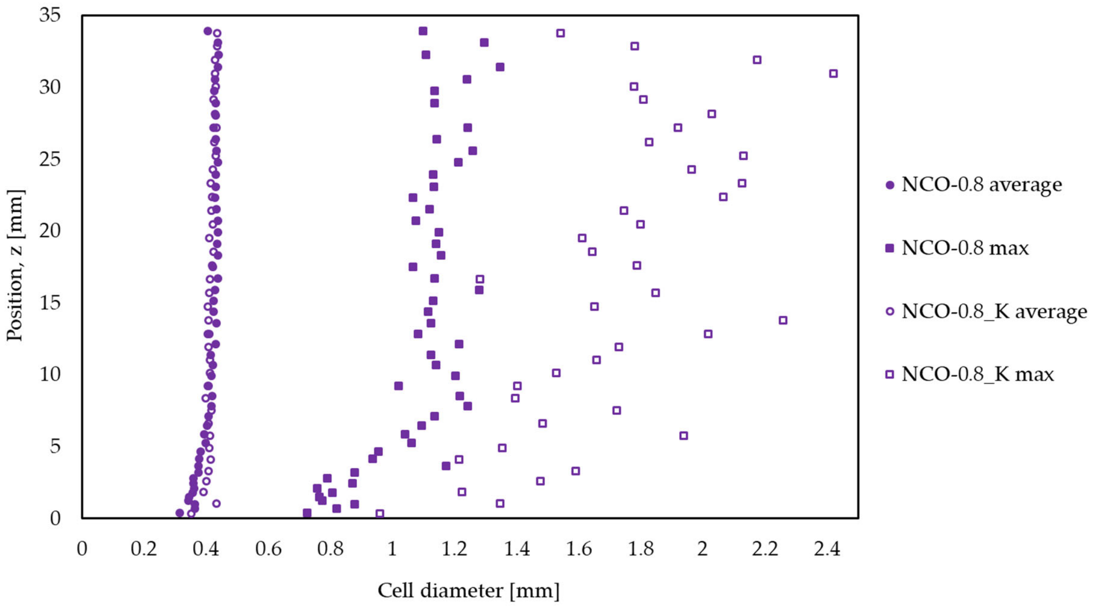

Figure S1 Average and maximum cell diameters along the sample height of the NCO-0.8 sample examined with a micro-CT before (NCO-0.8, filled circles and squares) and after (NCO-0.8_K, empty circles and squares) dry heat aging.

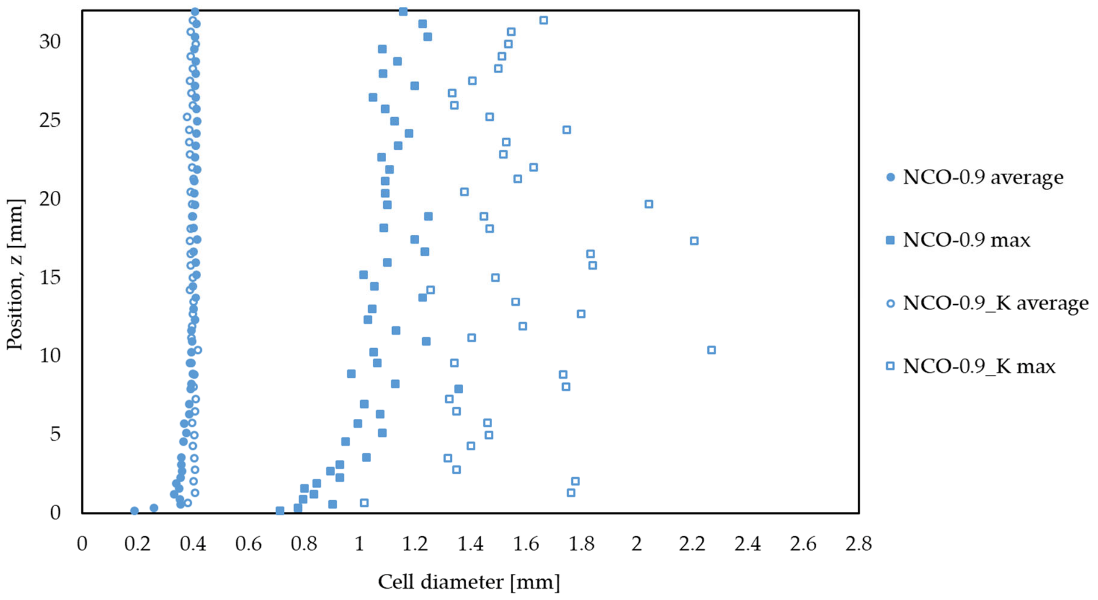

Figure S2 Average and maximum cell diameters along the sample height of the NCO-0.9 sample examined with a micro-CT before (NCO-0.9, filled circles and squares) and after (NCO-0.9_K, empty circles and squares) dry heat aging.

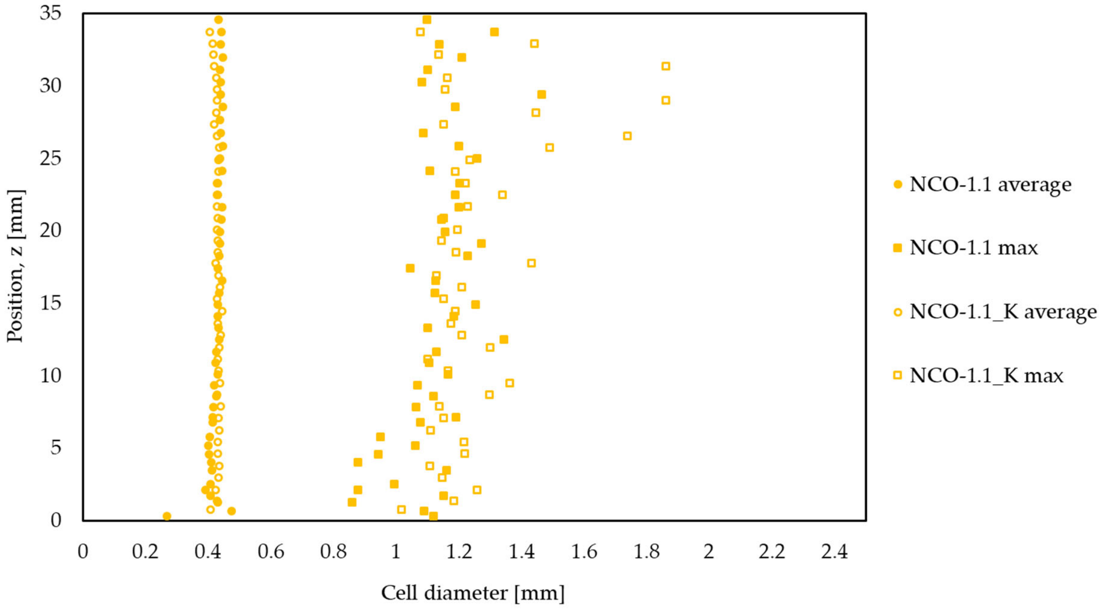

Figure S3 Average and maximum cell diameters along the sample height of the NCO-1.1 sample examined with a micro-CT before (NCO-1.1, filled circles and squares) and after (NCO-1.1_K, empty circles and squares) dry heat aging.

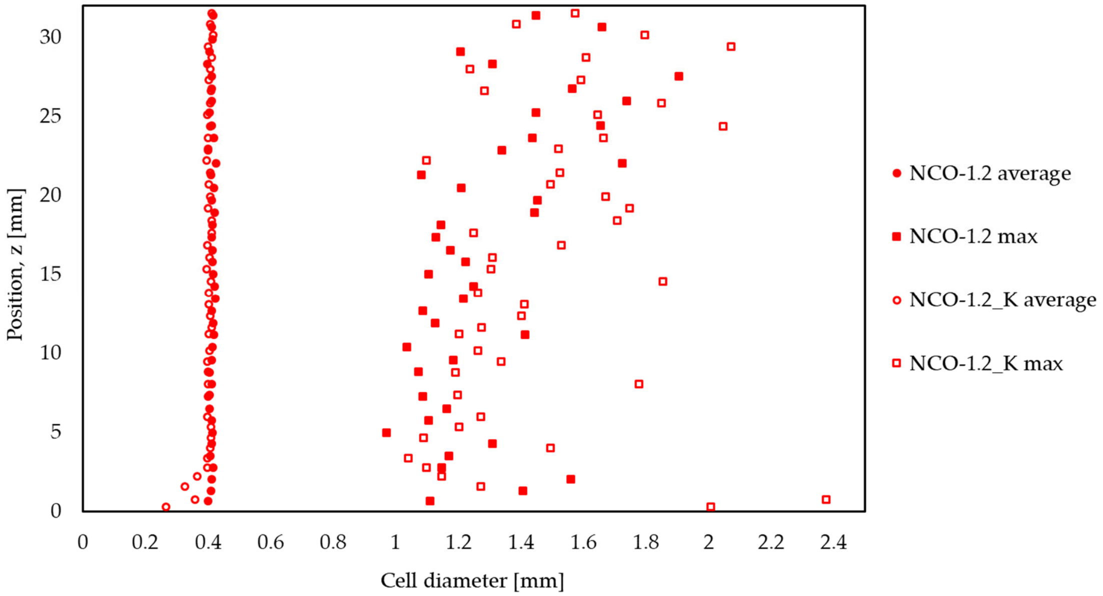

Figure S4 Average and maximum cell diameters along the sample height of the NCO-1.2 sample examined with a micro-CT before (NCO-1.2, filled circles and squares) and after (NCO-1.2_K, empty circles and squares) dry heat aging.
